# Identification of Olfactory Genes From the Greater Wax Moth by Antennal Transcriptome Analysis

**DOI:** 10.3389/fphys.2021.663040

**Published:** 2021-05-19

**Authors:** Xing-Chuan Jiang, Su Liu, Xiu-Yun Jiang, Zheng-Wei Wang, Jin-Jing Xiao, Quan Gao, Cheng-Wang Sheng, Teng-Fei Shi, Hua-Rui Zeng, Lin-Sheng Yu, Hai-Qun Cao

**Affiliations:** ^1^Anhui Provincial Key Laboratory of Integrated Pest Management on Crops, School of Plant Protection, Anhui Agricultural University, Hefei, China; ^2^CAS Key Laboratory of Tropical Forest Ecology, Xishuangbanna Tropical Botanical Garden, Chinese Academy of Sciences, Kunming, China

**Keywords:** *Galleria mellonella*, antenna, transcriptome, olfactory genes, expression pattern, genomic distribution

## Abstract

The olfactory system is used by insects to find hosts, mates, and oviposition sites. Insects have different types of olfactory proteins, including odorant-binding proteins (OBPs), chemosensory proteins (CSPs), odorant receptors (ORs), ionotropic receptors (IRs), and sensory neuron membrane proteins (SNMPs) to perceive chemical cues from the environment. The greater wax moth, *Galleria mellonella*, is an important lepidopteran pest of apiculture. However, the molecular mechanism underlying odorant perception in this species is unclear. In this study, we performed transcriptome sequencing of *G. mellonella* antennae to identify genes involved in olfaction. A total of 42,544 unigenes were obtained by assembling the transcriptome. Functional classification of these unigenes was determined by searching against the Gene Ontology (GO), eukaryotic orthologous groups (KOG), and the Kyoto Encyclopedia of Genes and Genomes (KEGG) databases. We identified a total of 102 olfactory-related genes: 21 *OBP*s, 18 *CSP*s, 43 *OR*s, 18 *IR*s, and 2 *SNMP*s. Results from BLASTX best hit and phylogenetic analyses showed that most of the genes had a close relationship with orthologs from other Lepidoptera species. A large number of *OBP*s and *CSP*s were tandemly arrayed in the genomic scaffolds and formed gene clusters. Reverse transcription-quantitative PCR results showed that *GmelOBP19* and *GmelOR47* are mainly expressed in male antennae. This work provides a transcriptome resource for olfactory genes in *G. mellonella*, and the findings pave the way for studying the function of these genes.

## Introduction

Olfaction is essential for insect activities such as food seeking, mate recognition, and oviposition. For efficient detection of chemical cues, insects have evolved an olfaction system that consists of many olfactory proteins, including odorant-binding proteins (OBPs), chemosensory proteins (CSPs), odorant receptors (ORs), ionotropic receptors (IRs), and sensory neuron membrane proteins (SNMPs) (Leal, 2013; Robertson, 2019).

OBPs are small, water soluble proteins enriched in the sensillar lymph of insect antennae (Pelosi et al., 2018). OBPs in the pores of the antennal sensillae can bind odorant compounds and deliver them to active ORs (Sun et al., 2018). OBPs typically have six positionally conserved cysteine residues. These cysteine residues form three disulfide bridges, which are necessary for maintaining protein stability (Brito et al., 2016). In Lepidoptera, there are two special subgroups of OBP: general odorant-binding protein (GOBP) and pheromone-binding protein (PBP) (Vogt et al., 2015). GOBPs recognize “general” odorants such as volatiles from host plants, whereas PBPs perceive sex pheromone constituents. However, many studies have demonstrated that GOBPs can bind sex pheromones and PBPs can have strong affinities for plant volatiles (Gong et al., 2009b; Khuhro et al., 2017; Sun et al., 2019a). CSPs are carrier proteins enriched in the sensillar lymph with a function similar to OBPs (Pelosi et al., 2018). CSPs contain four positionally conserved cysteines that form two disulfide bridges (Pelosi et al., 2014). Some CSPs are specifically expressed in the antenna and can bind to plant volatiles and sex pheromone constituents (Zhang et al., 2014; Li et al., 2015; Duan et al., 2019). Other CSPs are highly concentrated in non-olfaction organs, such as pheromone glands and legs, suggesting they may be involved in other physiological processes besides being carriers of odorants (Zhang et al., 2016; Sun et al., 2017).

Insect ORs are located on the dendrite membrane of olfactory sensory neurons (OSNs) (Touhara and Vosshall, 2009). ORs can recognize the odorants transferred by OBPs and CSPs, and convert these chemical signals into electrical signals (Wicher, 2018). Although most insect ORs have a seven-transmembrane domain, they are not G-protein-coupled receptors (GPCRs) because they have a different type of topology (Fleischer et al., 2018). In insects, a functional OR unit comprised of one copy of poorly conserved, conventional OR along with one copy of a highly conserved, non-conventional olfactory co-receptor (Orco) (Touhara and Vosshall, 2009). The OR/Orco complex forms heteromeric ligand gated ion channels that allow insects to rapidly perceive chemical signals (Butterwick et al., 2018). IRs are also key receptors involved in the perception of odorants, such as phenylacetaldehyde, amines and acids (Rytz et al., 2013; Zhang et al., 2019). IRs are transmembrane proteins with an extracellular N-terminus, a bipartite ligand-binding domain (two lobes separated by an ion channel domain), and a short cytoplasmic C-terminus, which have a structural similarity with ionotropic glutamate receptors (iGluRs) (Benton et al., 2009). However, IRs and iGluRs diverge from each other according to their sequence characteristics and phylogenetics (Croset et al., 2010).

Insect SNMPs have homology with the human fatty acid transporter CD36 and are divided into two subfamilies: SNMP1 and SNMP2 (Vogt et al., 2009). SNMP1s are co-expressed with pheromone receptors (PRs) accumulating on the membrane of pheromone-sensitive OSNs, whereas SNMP2s are expressed in the cells surrounding the pheromone-sensitive OSNs (Forstner et al., 2008; Sun et al., 2019b). SNMPs may have the ability to transfer lipophilic sex pheromones to ORs; in fruit fly and several moth species, SNMP1s are crucial for the detection of pheromones (Jin et al., 2008; Zhang et al., 2020).

Identification of olfactory genes will help us understand the molecular mechanism of insect olfaction. This would be useful in developing novel environmentally friendly methods for pest management (Venthur and Zhou, 2018). For example, OBPs, CSPs, and ORs can be used to screen bioactive attractants and repellents and antagonists of Orco could inhibit insect olfactory behavior (Leal et al., 2008; Kepchia et al., 2017; Choo et al., 2018; Zeng et al., 2018). Knockdown and knockout of particular genes by RNA interference and CRISPR techniques, respectively, can effectively block the communication between pest insects and their hosts (Pelletier et al., 2010; Dong et al., 2017; Garczynski et al., 2017; Zhu et al., 2019).

The greater wax moth, *Galleria mellonella*, is a major pest of honeybees throughout the world (Kwadha et al., 2017). Female *G. mellonella* lay eggs within the beehive, and the larvae feed on the wax comb and honey. They cause heavy losses in the beekeeping industry (Zhu et al., 2016). Traditional methods for controlling *G. mellonella* are based on chemical insecticides, but these may cause pesticide contamination of honey products. Adult *G. mellonella* detect host volatiles and sex pheromone using olfactory adaptations (Payne and Finn, 1977; Li et al., 2019). The molecular mechanisms of olfaction are therefore important for identifying the key genes mediating chemical signal perception and developing RNAi-based management strategies. Zhao et al. (2019) analyzed the antennal transcriptome of *G. mellonella* and identified a number of chemosensory genes, including 22 OBPs, 20 CSPs, 46 ORs, 17 IRs, and 2 SNMPs. However, these numbers are fewer than the numbers found in other Lepidoptera species and suggest the existence of other, unidentified, genes.

In this study, we performed transcriptome sequencing of the *G. mellonella* antennae. We identified 102 olfactory-related genes, including 11 novel genes, from the transcriptome dataset. We analyzed the sequence characteristics, phylogeny, genomic distribution, and exon–intron organization of these genes. We also determined the expression profiles of the newly identified genes using reverse transcription-quantitative PCR (RT-qPCR).

## Materials and Methods

### Insects

The *G. mellonella* used in this study originated from a colony collected from infested beehives on a bee farm in Hefei, China. The larvae were reared on an artificial diet and the adults were fed on a 10% (v/v) honey solution. Insects were reared at 27°C ± 1°C, 65 ± 5% relative humidity and a photoperiod of 14:10 h (L:D).

### Sample Collection and RNA Extraction

Adult males and females (2-day-old, unmated) were sampled and different tissues were dissected. These included 300 male antennae, 300 female antennae, 60 heads (without antennae; 30 males and 30 females, pooled), 60 abdomens (30 males and 30 females, pooled), and 300 legs (150 males and 150 females, pooled). Total RNA was isolated using Trizol reagent (Life Technologies, Carlsbad, CA, United States) following manufacturer protocol. The integrity and concentration of the RNA was determined using agarose gel electrophoresis and spectrophotometry, respectively.

### cDNA Library Construction

Total RNA (20 μg) from male and female antennae were used to create cDNA libraries. In brief, poly(A)+ mRNA was purified from total RNA using oligo(dT) magnetic beads and was digested to short fragments in a fragmentation buffer. The fragmented mRNA was used to generate first-strand cDNA using a random hexamer primer and MMLV reverse transcriptase, and second-strand cDNA was subsequently synthetized in a mixture of DNA polymerase I, dNTPs and RNaseH. The double-stranded cDNA was treated with T4 DNA polymerase for end-repair and T4 polynucleotide kinase for dA-tailing. After ligation of the sequencing adapters with T4 DNA ligase, these fragments were used as templates for PCR amplification. Finally, the PCR product was heat-denatured and the single-stranded cDNA was cyclized by splint oligonucleotide and DNA ligase to generate the library.

### Transcriptome Assembly and Functional Annotation

The cDNA libraries from male and female antennae were sequenced on a BGISEQ-500 system using a paired-end sequencing method according to manufacturer instructions at the Beijing Genomics Institute (BGI-Wuhan, Wuhan, China). Before *de novo* assembly, the adapters and low-quality reads were filtered, and removed, from the raw data. Clean reads from males and females were assembled into a single assembly using Trinity software (v2.0.6; Grabherr et al., 2011). Reads were combined to form contigs, from which scaffolds were extended by paired-end joining and gap-filling. If a scaffold could not be extended on either end, it was defined as a unigene. Functional annotation of each unigene was performed using the BLASTX program against the NCBI non-redundant (NR) database, Gene Ontology (GO), and eukaryotic orthologous groups (KOG) with a cut-off *e*-value of 10^–5^. The Kyoto Encyclopedia of Genes and Genomes (KEGG) pathways annotations were performed using the KEGG automatic annotation server (Yoshizawa et al., 2007).

### Identification of Olfactory Genes

Candidate olfactory genes were identified by retrieving the transcriptome dataset with the TBLASTN program (Altschul et al., 1997). The annotated protein sequences of OBPs, CSPs, ORs, IRs, and SNMPs from other Lepidoptera species, including *Bombyx mori*, *Plutella xylostella*, *Manduca sexta*, *Helicoverpa armigera*, *Spodoptera litura*, *Chilo suppressalis*, *Cnaphalocrocis medinalis*, and *Ostrinia furnacalis*, were used as queries. The cut-off *e*-value was set as 10^–5^. The output was manually checked, and overlapping variants were eliminated. Finally, all the candidates were confirmed by searching against the NCBI NR database using the BLASTX online program^1^ (cut-off *e*-value: 10^–5^). In addition, all the candidate genes were compared with those reported by Zhao et al. (2019) using BLASTN program (cut-off *e*-value: 10^–5^; Altschul et al., 1997), in order to find novel olfactory genes in *G. mellonella*.

### Bioinformatic Analyses

The open reading frame (ORF) was predicted using ORF Finder^2^. The theoretical molecular weight (Mw) and isoelectric point (pI) were obtained using an ExPASy tool^3^. Putative signal peptide and the transmembrane domain were predicted with SignalP^4^ and TMHMM^5^, respectively. The Clustal Omega program^6^ was used to align deduced protein sequences. The phylogenetic trees were constructed with MEGA7 software using the neighbor-joining method with 1,000-fold bootstrap resampling (Kumar et al., 2016). The trees were viewed and edited using FigTree software^7^. The GenBank accession numbers of sequences used in the phylogenetic analyses are listed in Supplementary Table 1. Motif pattern analysis was performed using the MEME program^8^; insect OBPs and CSPs used in the analysis are listed in Supplementary Table 2. The genomic distribution of each gene was determined by mapping the cDNA with the *G. mellonella* genomic DNA (Lange et al., 2018) using the Splign program^9^.

### RT-qPCR

Total RNA from different adult tissues (see “Sample Collection and RNA Extraction” section) was reverse transcribed to generate first-strand cDNA using ReverTra Ace qPCR RT Master Mix with gDNA Remover (Toyobo, Osaka, Japan). Each cDNA sample was diluted to 10 ng/μL using nuclease-free water. RT-qPCR was performed in a 20 μL reaction mixture containing 10 μL SYBR Green Real-time PCR Master Mix (Toyobo, Osaka, Japan), 1 μL (10 ng) cDNA template, 0.4 μL (0.2 μM) of forward primer, 0.4 μL (0.2 μM) of reverse primer, and 8.2 μL nuclease-free water. Primers for RT-qPCR are listed in Supplementary Table 3, and the glyceraldehyde-3-phosphate dehydrogenase (*GAPDH*) gene was used as an internal reference to normalize target gene expression. RT-qPCR reactions were conducted in 96-well plates and run on a CFX96 Real-time System (Bio-Rad, Hercules, CA, United States). The thermal cycle parameters were one cycle of 95°C for 2 min, 40 cycles of 95°C for 5 s, and 60°C for 20 s. At the end of each thermal cycle, the PCR products were analyzed using a heat-dissociation protocol to confirm that only one single gene was amplified. A no-template control and a no-reverse transcriptase control were both included in each reaction plate to detect possible contamination. The experiment was biologically repeated three times (each with four technical replicates). Relative expression levels were calculated by using the 2^–ΔΔCt^ method (Livak and Schmittgen, 2001).

### Statistics

Data were analyzed using Data Processing System (DPS) software version 9.5 (Tang and Zhang, 2013). One-way analysis of variance (ANOVA) with Tukey’s *post hoc* test was performed to analyze differences of gene expression levels among multiple samples. Comparisons were considered significant at a *p* < 0.05.

## Results

### Transcriptome Sequencing and Unigene Assembly

**FIGURE 1:**
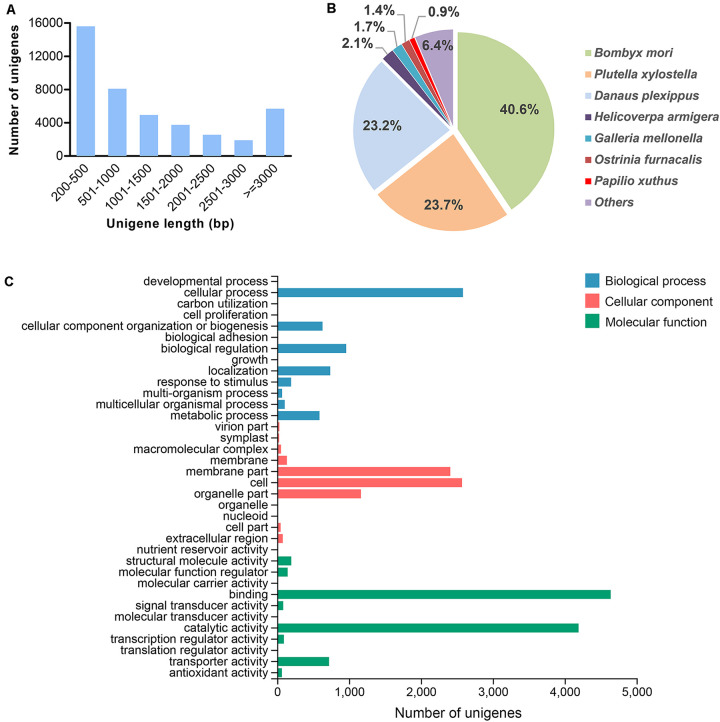
Summary of the *G. mellonella* unigene assembly. **(A)** Size distribution of unigenes. **(B)** Species distribution of unigenes based on homology searching against the NCBI NR database. **(C)** GO classification of unigenes.

In total, 73.8 and 73.9 Mb raw reads were generated from the transcriptomes of male and female antennae, respectively (Table 1). These data have been deposited into the NCBI Sequence Read Archive (SRA) database under accession numbers SRR8307568 (male antennae) and SRR8307567 (female antennae). After data filtration, 70.3 Mb (male antennae) and 70.6 Mb (female antennae) clean reads were obtained. Clean reads from the two transcriptomes were assembled into 42,544 unigenes (Table 1). The size distribution analysis showed that the lengths of 18,844 unigenes (44.3% of all unigenes) were greater than 1,000 bp (Figure 1A).

**TABLE 1:** Information of the *G. mellonella* antennal transcriptome.

	**Male antennae**	**Female antennae**
Total size	7.0 Gb	7.0 Gb
Total number of raw reads	73.8 Mb	73.9 Mb
Total number of clean reads	70.3 Mb	70.6 Mb
Q20 (%)	97.4	97.4
Total number of unigenes	42,544	
Total length of unigenes	60.4 Mb	
Maximum length of unigenes	21,594 bp	
Mean length of unigenes	1,420 bp	
Minimum length of unigenes	297 bp	
N50	2,617 bp	
GC (%)	38.4	

### Functional Annotation

We annotated the *G. mellonella* unigenes by searching against the NCBI NR database. A total of 14,481 (34%) of the 42,544 unigenes resulted from the search (Figure 1B). For species distribution, the *G. mellonella* unigenes were best matched to those from other species of Lepidoptera, including *B. mori* (40.6%), *P. xylostella* (23.7%), and *Danaus plexippus* (23.2%) (Figure 1B). Next, we performed a GO analysis to better classify the functions of the *G. mellonella* unigenes. The results indicated that 8,887 (20.9%) of the unigenes could be annotated to at least one GO term (Figure 1C). Among the GO categories, the *G. mellonella* unigenes were mostly enriched in “binding” and “catalytic activity” categories in the “molecular function” level, followed by “cell” and “membrane part” categories in the “cellular component” level, and “cellular process” category in the “biological process” level (Figure 1C). We also performed the functional classification for the unigenes by searching against KOG and KEGG databases, and the results are shown in Supplementary Figures 1, 2, respectively.

### Identification of *OBPs*

Zhao et al. (2019) identified 22 *OBP*s from *G. mellonella* antennae, including 2 *GOBP*s, 3 *PBP*s, and 17 *OBP*s. In the present study, we screened the antennal transcriptome dataset and identified 21 genes (Supplementary Table 4). Of these, four (*GmelOBP18* to *GmelOBP21*) are novel genes. A comprehensive list of *G. mellonella* OBPs is shown in Supplementary Figure 3, and the nucleotide and amino acid sequences of genes identified are listed in Supplementary Table 5. We found at least 26 *OBP*s expressed in the antennae of *G. mellonella*. Of the identified OBPs, 16 sequences had complete ORFs, while *GmelOBP4, GmelOBP20*, and *GmelOBP21* lacked the 5′- and/or 3′-terminus (Supplementary Table 4). Most of the *OBP*s shared ≥51% amino acid identities with orthologs from other Lepidoptera species, whereas three OBPs, *GmelOBP8*, *GmelOBP13*, and *GmelOBP18*, shared 28, 47, and 38% amino acid identities, respectively, with their respective orthologs (Supplementary Table 4).

**FIGURE 2:**
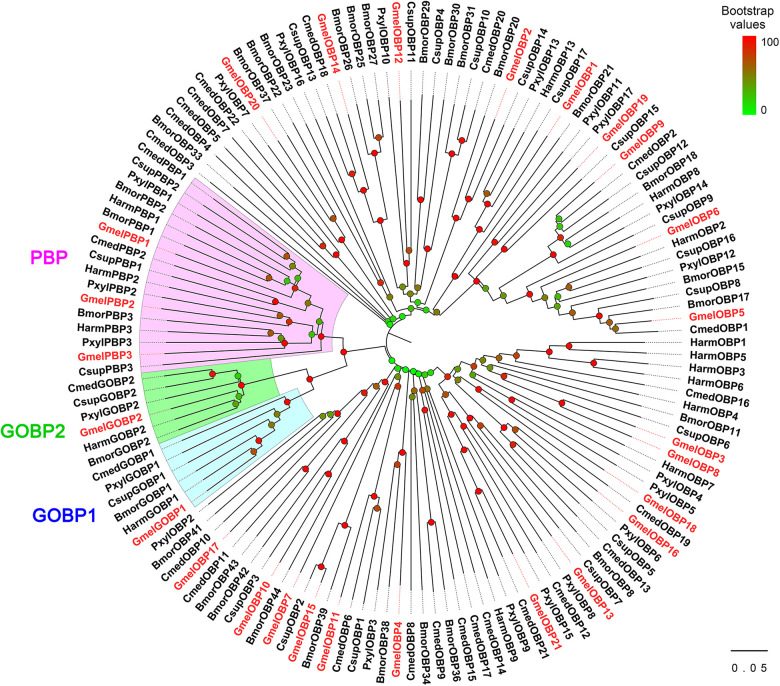
Phylogenetic analysis of OBPs from *G. mellonella* (Gmel-prefix) and other lepidopterans, including *Bombyx mori* (Bmor), *Plutella xylostella* (Pxyl), *Helicoverpa armigera* (Harm), *Chilo suppressalis* (Csup), and *Cnaphalocrocis medinalis* (Cmed). Bootstrap values are indicated by colors from green (0) to red (100). The *G. mellonella OBP*s are highlighted in red. GenBank accession numbers of genes are listed in Supplementary Table 1.

**FIGURE 3:**
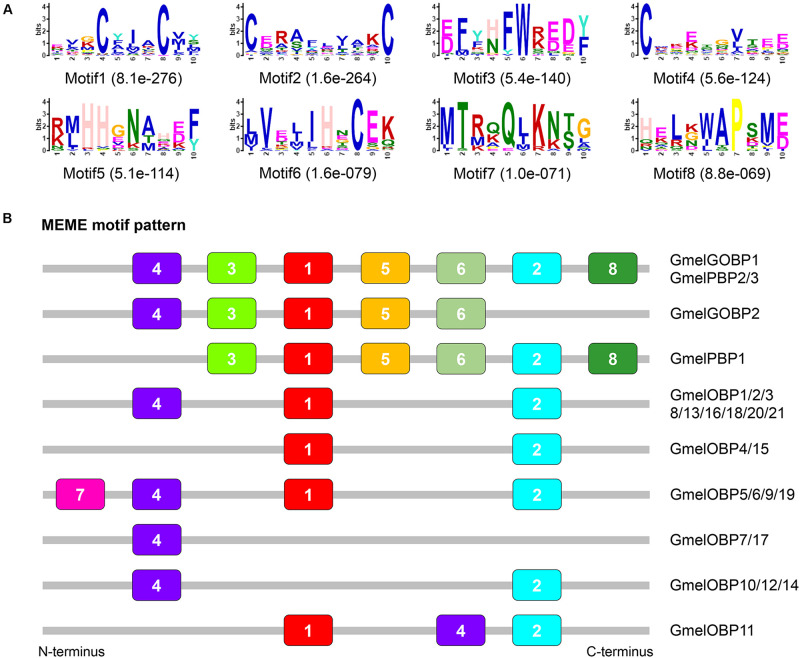
Motif pattern analysis of *G. mellonella* OBPs. **(A)** The discovered eight motifs (motif1–8) in GmelOBP proteins and their homologs from other lepidopterans. The number in the parentheses indicated the expect-value (*e*-value) of each motif calculated by MEME program. **(B)** Locations of each motif in the protein sequences. The numbers in the colored boxes correspond to the numbered motifs in **(A)**. The protein sequences of the OBPs used are listed in Supplementary Table 2.

The multiple sequence alignment result showed that six positionally conserved cysteine residues were presented in all OBP proteins except for GmelOBP14, which only had four cysteine residues (Supplementary Figure 4). A phylogenetic tree was constructed and the results indicated that the *G. mellonella* OBPs were well-segregated from each other with high bootstrap support; most of them were clustered with at least one lepidopteran ortholog (Figure 2). We used the MEME program to investigate the motif patterns in the identified OBPs and found eight conserved motifs (Figure 3A). GmelGOBP1, GmelPBP2, and GmelPBP3 have the same motif pattern 4-3-1-5-6-2-8; GmelGOBP2 is similar to GmelGOBP1 but lacks the seventh and eighth motifs at its C-terminus, whereas GmelPBP1 lacks the first motif at the N-terminus (Figure 3B). The most conserved motif pattern (4-1-2) was observed in nine OBPs (GmelOBP1/2/3/8/13/16/18/20/21), whereas GmelOBP7 and GmelOBP17 only had the fourth motif (Figure 3B).

### Identification of *CSPs*

A total of 18 *CSP* genes were retrieved from the transcriptome dataset (Supplementary Tables 4, 5). All of these *CSP*s had complete ORFs and the length of the deduced proteins ranged from 97 to 131 amino acids (Supplementary Table 4). BLASTX best hit results showed that three *CSP*s (*GmelCSP9*, *GmelCSP12*, and *GmelCSP13*) had low amino acid identities (33–42%) to other known *CSP*s, whereas the other 15 CSPs had high amino acid identities (61–85%) to their lepidopteran orthologs (Supplementary Table 4).

**FIGURE 4:**
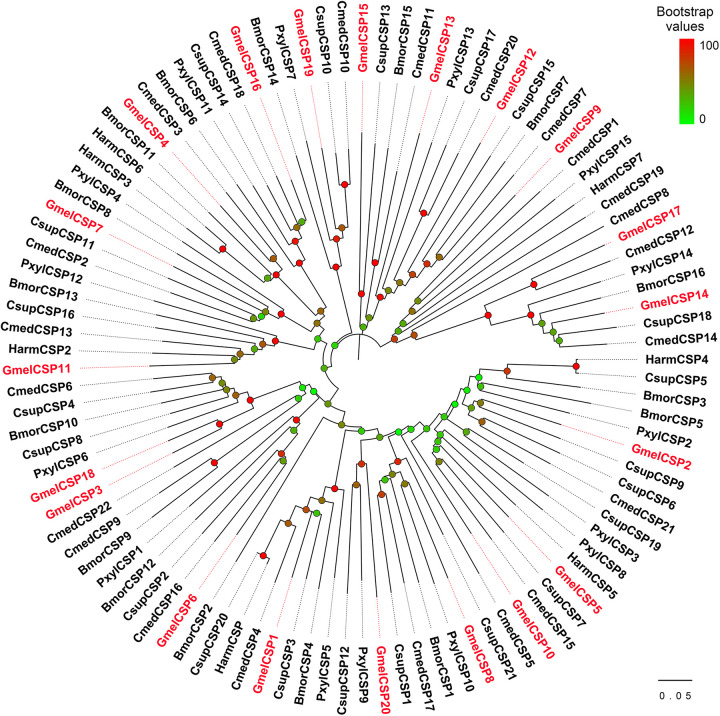
Phylogenetic analysis of CSPs from *G. mellonella* (Gmel-prefix) and other lepidopterans, including *Bombyx mori* (Bmor), *Plutella xylostella* (Pxyl), *Helicoverpa armigera* (Harm), *Chilo suppressalis* (Csup), and *Cnaphalocrocis medinalis* (Cmed). Bootstrap values are indicated by colors from green (0) to red (100). The *G. mellonella CSP*s are highlighted in red. GenBank accession numbers of genes are listed in Supplementary Table 1.

**FIGURE 5:**
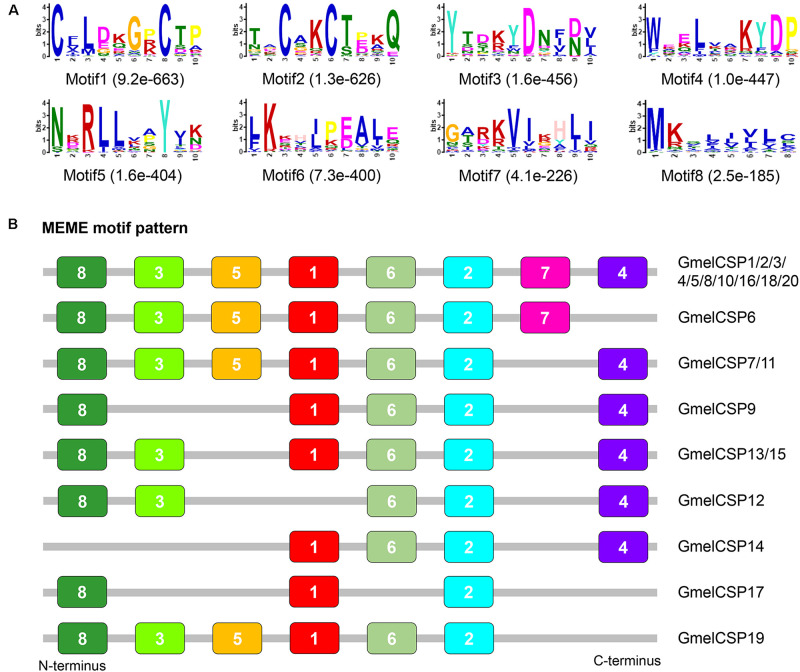
Motif pattern analysis of *G. mellonella* CSPs. **(A)** Eight motifs (motif 1–8) discovered in GmelCSP proteins and their lepidopteran homologs. The number in the parentheses indicated the expect-value (*e*-value) calculated by MEME program. **(B)** Locations of each motif in the protein sequences. The numbers in the colored boxes correspond to the numbered motifs in **(A)**. The CSP protein sequences used are listed in Supplementary Table 2.

Multiple sequence alignment showed that all the deduced GmelCSP proteins had four positionally conversed cysteines (Supplementary Figure 5). Phylogenetic analysis showed that, like GmelOBPs, most GmelCSPs were spread across different branches and that they were clustered with at least one lepidopteran ortholog (Figure 4). The MEME program revealed that the motif pattern 8-3-5-1-6-2-7-4 is most conserved, which existed in 10 (GmelCSP1/2/3/4/5/8/10/16/18/20) of the 20 CSPs (Figure 5). GmelCSP7 and GmelCSP11 had the motif pattern 8-3-5-1-6-2-4, and GmelCSP13 and GmelCSP15 had the pattern 8-3-1-6-2-4. Other GmelOBPs had distinct patterns (Figure 5).

### Identification of *ORs*

**FIGURE 6:**
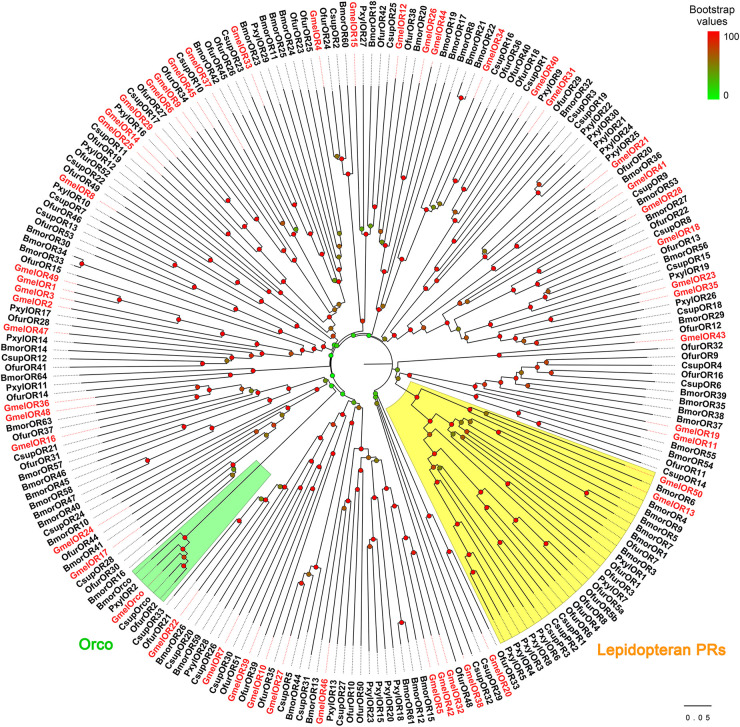
Phylogenetic relationships of lepidopteran ORs. Gmel: *Galleria mellonella*; Bmor: *Bombyx mori*; Ofur: *Ostrinia furnacalis*; Csup: *Chilo suppressalis*; Pxyl: *Plutella xylostella*. Bootstrap values are indicated by colors from green (0) to red (100). The *G. mellonella OR*s are highlighted in red. GmelOR30 was not used for the analysis due to its short length. GenBank accession numbers of ORs used are listed in Supplementary Table 1.

We identified 43 putative *OR*s from the transcriptome (Supplementary Tables 4, 5). Of these, five (*GmelOR46* to *GmelOR50*) were novel genes, and other sequences were previously identified by Zhao et al. (2019) (Supplementary Figure 3). The total number of *GmelOR*s is expected to reach 51. Of the *OR*s, 27 sequences had complete ORFs, whereas other sequences had truncations in the 5′- and/or 3′-ternimus (Supplementary Table 4). The length of the deduced OR proteins ranged from 163 to 474 amino acids, and the transmembrane domains were predicted in all the OR proteins (Supplementary Table 4). BLASTX best hit results showed that all the *GmelOR*s had orthologs in other species of Lepidoptera, including *O. furnacalis*, *Amyelois transitella*, *H. armigera*, and *B. mori* (Supplementary Table 4). In the phylogenetic analysis, GmelORs were well-segregated from each other with high bootstrap support, and most of them were clustered with at least one lepidopteran ortholog (Figure 6). As expected, the olfactory co-receptor, *GmelOrco*, was clustered into a branch with *Orco*s from *C. suppressalis*, *O. furnacalis*, *P. xylostella*, and *B. mori* (Figure 6). Additionally, *GmelOR13* and *GmelOR50* fell into the “Lepidopteran pheromone receptors (PRs)” clade with PRs from other Lepidoptera species, e.g., BmorOR1 and BmorOR3 from *B. mori*; PxylOR1 and PxylOR4 from *P. xylostella* (Figure 6).

### Identification of *IRs* and *SNMPs*

**FIGURE 7:**
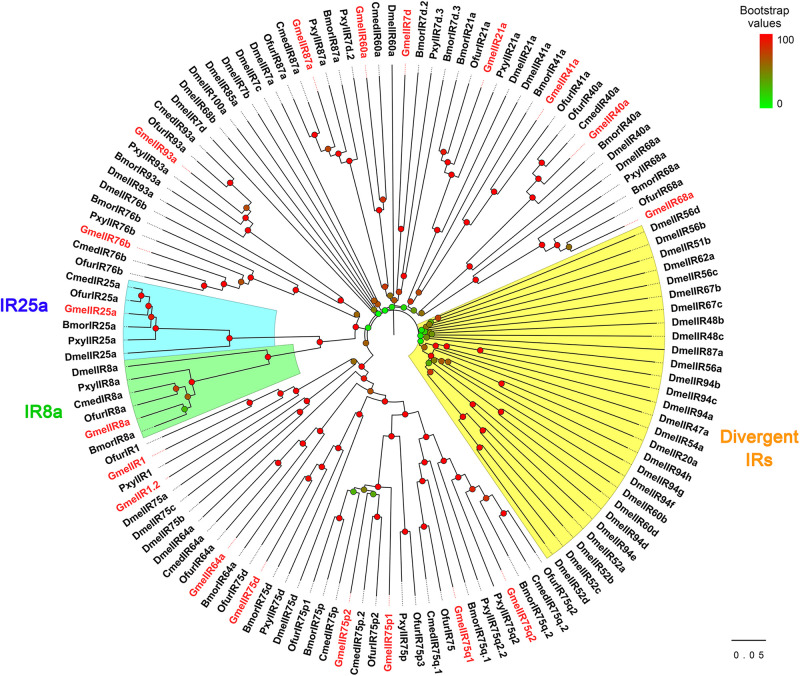
Phylogenetic relationships of insect IRs. Dmel: *Drosophila melanogaster*; Bmor: *Bombyx mori*; Ofur: *Ostrinia furnacalis*; Pxyl: *Plutella xylostella*; Cmed: *Cnaphalocrocis medinalis*. Bootstrap values are indicated by colors from green (0) to red (100). The *G. mellonella* IRs are highlighted in red. GenBank accession numbers of IRs used are listed in Supplementary Table 1.

We identified 18 putative *IR*s, including two novel genes (Supplementary Tables 4, 5). Together with the results of Zhao et al., the expected number of *IR*s in *G. mellonella* antennae is at least 19 (Supplementary Figure 3). Of these, 15 *IR*s had complete ORFs and the length of the deduced proteins ranged from 451 to 931 amino acid residues. All of the GmelIRs were transmembrane proteins which contained 2–4 transmembrane domains (Supplementary Table 4). Most of the GmelIRs shared ≥52% amino acid identities with their respective orthologs from other lepidopterans except for GmelIR7d and GmelIR75q1, which shared 45 and 48% amino acid identities, respectively, with other insect IRs (Supplementary Table 4). Phylogenetic analysis showed that most of the GmelIRs were segregated from each other, and that most GmelIRs were located on branches with other lepidopteran IRs (Figure 7). In addition, two putative co-receptors, GmelIR8a and GmelIR25a, were also identified (Supplementary Table 4 and Figure 7).

**FIGURE 8:**
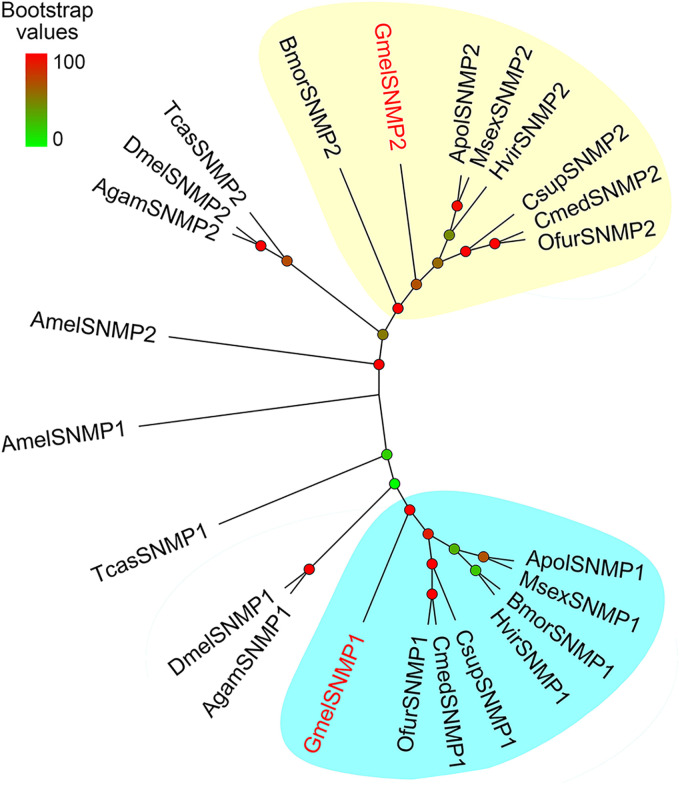
Phylogenetic relationships of insect SNMPs. Gmel: *Galleria mellonella*; Apol: *Antheraea polyphemus*; Bmor: *Bombyx mori*; Csup: *Chilo suppressalis*; Cmed: *Cnaphalocrocis medinalis*; Hvir: *Heliothis virescens*; Msex: *Manduca sexta*; Ofur: *Ostrinia furnacalis*; Agam: *Anopheles gambiae*; Amel: *Apis mellifera*; Dmel: *Drosophila melanogaster*; Tcas: *Tribolium castaneum*. Bootstrap values are indicated by colors from green (0) to red (100). The *G. mellonella* SNMPs are highlighted in red. GenBank accession numbers of genes used are listed in Supplementary Table 1.

We identified two *SNMP*s in *G. mellonella*, namely, *GmelSNMP1* and *GmelSNMP2*. *GmelSNMP1* shared 71% amino acid identity with *SNMP1* in *Eogystia hippophaecolus*, while *GmelSNMP2* was more similar to the *O. nubilalis* SNMP2 (66% amino acid identity) (Supplementary Table 4). The two deduced GmelSNMP proteins both had two transmembrane domains, and had five positionally conserved cysteine residues (Supplementary Figure 6). Phylogenetic analysis showed that *GmelSNMP1* and *GmelSNMP2* had a close relationship with their lepidopteran orthologs (Figure 8).

### Genomic Localization of Olfactory Genes

**FIGURE 9:**
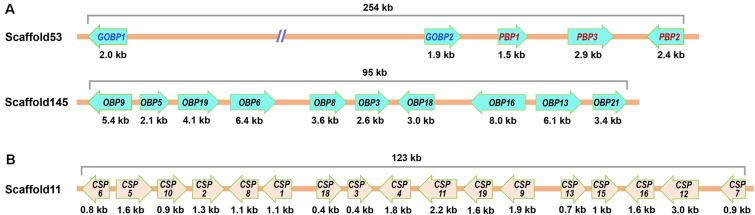
Genomic location of *OBP*s **(A)** and *CSP*s **(B)** in *G. mellonella*.

We determined the genomic distribution of the olfactory genes identified from *G. mellonella* by mapping the cDNA sequences to genome scaffolds. We successfully matched the 118 genes (containing 16 genes identified by Zhao et al., 2019) to 61 scaffolds (Supplementary Table 6). Of the 26 *OBP*s, 2 *GmelGOBP*s, and 3 *GmelPBP*s were located on scaffold53, while another 10 *OBP*s (*GmelOBP3*/*5*/*6*/*8*/*9*/*13*/*16*/*18*/*19*/*21*) were tandemly arrayed on scaffold145 (Figure 9A and Supplementary Table 6). Of the 20 *CSP*s, 17 were found to be clustered within a 123 kb genomic region on scaffold11 (Figure 9B and Supplementary Table 6).

For *OR*s, we found that most of the scaffolds contained only one or two *OR* genes; the exceptions were scaffold2, scaffold42, scaffold43, scaffold611, and scaffold681, each of which contained three *GmelOR*s (Supplementary Table 6). For *IR*s and *SNMP*s, we mapped *GmelIR75p1* and *GmelIR75p2* on scaffold319, and *GmelIR75q1* and *GmelIR75q2* on scaffold172 (Supplementary Table 6). The remaining *IR*s, as well as the *SNMP*s, were located individually on a single scaffold (Supplementary Table 6).

### Expression Profiles of Olfactory Genes

**FIGURE 10:**
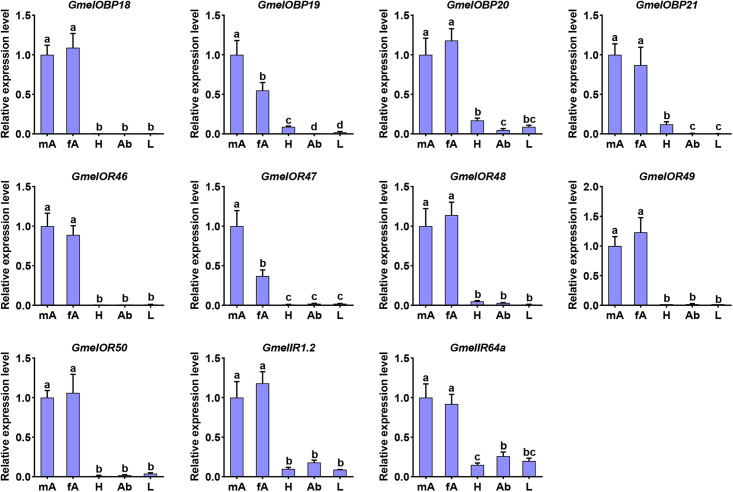
Expression profiles of olfactory genes in different adult tissues. mA: male antennae, fA: female antennae, H: head (without antennae), Ab: abdomen, L: legs. Gene expression levels in various tissues were normalized relative to that in male antennae (set as onefold). Data are presented as mean (*n* = 3) ± SE. Different lowercase letters indicate significant differences (*p* < 0.05, one-way ANOVA with Tukey’s test).

The tissue- and sex-biased expression profiles of the newly identified genes (four *OBP*s, five *OR*s, and two *IR*s) were investigated using RT-qPCR. All the tested genes were predominantly or highly expressed in the antennae (Figure 10). Of these, the transcripts of *GmelOBP19* and *GmelOR47* were enriched in male antennae, with expression levels 1.8-fold (*GmelOBP19*) and 2.7-fold (*GmelOR47*) higher in males than in females, respectively (Figure 10). Other genes were expressed at equal or near-equal amounts in the antennae of both sexes (Figure 10).

## Discussion

We constructed a transcriptome dataset from the *G. mellonella* antennae. Zhao et al. (2019) previously identified 22 *OBP*s, 20 *CSP*s, 46 *OR*s, 17 *IR*s, and 2 *SNMP*s in *G. mellonella* antennae. Here, we discovered 102 olfactory-related genes, including 11 novel genes. Our findings, together with the results of Zhao et al. (2019), provide a comprehensive data resource for the olfactory genes in *G. mellonella*.

We identified 21 *OBP*s, including four novel genes, in *G. mellonella* antennae. Therefore, the total number of *OBP*s in the *G. mellonella* antennae is at least 26. Although this number is lower than the number in *Drosophila melanogaster* (52 genes), *M. sexta* (49 genes), *Spodoptera littoralis* (49 genes), and *B. mori* (46 genes) (Gong et al., 2009a; Vieira and Rozas, 2011; Vogt et al., 2015; Walker et al., 2019), it is comparable to those from other Lepidoptera, such as *O. furnacalis* (23 genes), *S. exigua* (24 genes); *P. xylostella* (24 genes), *S. frugiperda* (25 genes), and *C. suppressalis* (26 genes) (Cao et al., 2014; Zhang et al., 2015, 2018; Yang et al., 2017; Qiu et al., 2020). A number of *OBP* genes are specifically expressed in non-olfactory tissues such as abdomen and legs, as well as in larval stages of other insect species (Hull et al., 2014; Vogt et al., 2015). Since we only sequenced the antennal transcriptome of *G. mellonella*, some *OBP* genes might have been missed in the present research. Further studies examining additional tissues and developmental stages are needed.

The number of *CSP* genes in insect genomes appears to be highly variable. For instance, 34 and 33 *CSP*s were found in lepidopterans *D. plexippus* and *Heliconius melpomene*, respectively, whereas only four were discovered in the dipteran *D. melanogaster* (Vieira and Rozas, 2011; The Heliconius Genome Consortium, 2012). In this study, we identified 18 *CSP*s in *G. mellonella* antennae. The number of *GmelCSP*s is expected to reach 20 when combined with the genes discovered by Zhao et al. (2019). This number is less than the number in *D. plexippus* (34 genes) and *H. melpomene* (33 genes), but comparable to those identified in other lepidopterans, including *S. exigua* (19 genes; Zhang et al., 2018), *Plodia interpunctella* (15 genes; Jia et al., 2018), and *Streltzoviella insularis* (12 genes; Yang et al., 2019).

The motif pattern varies in different OBP and CSP proteins in insects (Zhang et al., 2016; Sun et al., 2017). Within *G. mellonella* OBPs, the most conserved pattern is 4-1-2, whereas 8-3-5-1-6-2-7-4 is most conserved in CSPs. This result implies a conserved function of the two protein families in odor recognition. We found that two GmelGOBPs and three GmelPBPs displayed different motif patterns: GmelGOBP2 lost the seventh and eighth motifs, and GmelPBP1 lacks the first motif, when compared with those in GmelGOBP1, GmelPBP2, and GmelPBP3 (Figure 3B). This difference suggests a possible functional differentiation. Indeed, a number of studies have indicated that lepidopteran GOBPs and PBPs display different affinities to odorants (Liu et al., 2015; Zhang et al., 2017).

We identified 43 *OR*s from *G. mellonella*, including 5 novel genes. The total number (51 genes) of *OR*s in *G. mellonella* is less than the 66 and 73 genes identified, respectively, in the genomes of *B. mori* and *S. litura*, two model lepidopteran insect species (Tanaka et al., 2009; Cheng et al., 2017), but comparable to those in *P. xylostella* (54 genes; Yang et al., 2017), *O. furnacalis* (52 genes; Yang et al., 2015), and *M. sexta* (48 genes; Grosse-Wilde et al., 2011). Numerous studies have reported that a subset of *OR* genes in insects have higher transcription levels in non-olfactory tissues than in antennae (Fleischer et al., 2018). Thus, our sequencing of the antennae limits our ability to identify potential *OR* genes enriched in other non-olfactory organs. We also identified *GmelOrco*, the olfactory co-receptor, from *G. mellonella*. Insect Orco is an essential component for forming a functional OR unit (Leal, 2013). Therefore, the identification of *GmelOrco* greatly benefits the development of synthetic inhibitors or genome-editing approaches to control this insect pest (Koutroumpa et al., 2016; Kepchia et al., 2017; Liu et al., 2017a).

Apart from *OR*s, 18 *IR*s were identified in our transcriptome search. In Lepidoptera, 17, 18, 21, and 21 *IR*s were found in the antennae of *S. littoralis*, *B. mori*, *H. armigera*, and *O. furnacalis*, respectively (Croset et al., 2010; Poivet et al., 2013; Liu et al., 2014; Yang et al., 2015). Thus, the *IR* gene number in *G. mellonella* antennae is comparable to those in other Lepidoptera. We also identified the orthologs (*GmelIR8a* and *GmelIR25a*) of the highly conserved co-receptors *IR8a* and *IR25a*. The two genes are expected to encode functional proteins and play a central role in forming a functional IR receptor complex (Abuin et al., 2011, 2019). The *M. sexta* IR8a is required for acid detection and is involved in the avoidance of acids from caterpillar feces (Zhang et al., 2019).

We identified two *SNMP*s (*GmelSNMP1* and *GmelSNMP2*) in *G. mellonella*. Previous research on *Heliothis virescens* and *Antheraea polyphemus* demonstrated that *SNMP1*s are co-expressed with *PR*s in the pheromone-responsive neurons, whereas *SNMP2*s are expressed in the supporting cells around the neurons (Forstner et al., 2008). Two *SNMP*s have distinct expression patterns in the antennal sensilla of *Ectropis obliqua* (Sun et al., 2019b), suggesting a functional diversification between the two genes. In *D. melanogaster*, *H. virescens*, and *B. mori*, SNMP1s play critical roles in pheromone signaling (Jin et al., 2008; Pregitzer et al., 2014; Zhang et al., 2020). The two *GmelSNMP*s identified here showed very high identities with orthologs in other insect species, indicating functional conservation among these proteins.

We analyzed the genomic distribution of olfactory genes in *G. mellonella* and found that a large number of *OBP*s and *CSP*s were located on the same scaffolds and formed gene clusters. Two or more *OBP* or *CSP* loci located on the same scaffold implies that they were derived through duplication events during evolution (Vieira and Rozas, 2011; Vogt et al., 2015). It is possible that the *G. mellonella OBP* and *CSP* families evolved through gene duplication. Clusters of *OBP* and *CSP* genes on the same scaffold have also been found in the genomes of many other insect species including *D. melanogaster*, *Apis mellifera*, *Anopheles gambiae*, and *B. mori* (Hekmat-Scafe et al., 2002; Forêt and Maleszka, 2006; Gong et al., 2009a). Further analysis of *OBP* or *CSP* gene duplication events in *G. mellonella* is needed and will extend our knowledge of gene evolution. *G. mellonella* adults display a unique pair-forming behavior in which the sex pheromone is produced by males and perceived by conspecific females (Kwadha et al., 2017). Hence, olfactory genes that are primarily expressed in female antennae might be involved in recognizing sex pheromone constituents. Previously, Zhao et al. (2019) identified several female antennae-biased genes and hypothesized that they may contribute to pheromone detection. In this study, we analyzed the expression profiles of the newly identified genes. However, we were unable to identify female antennae-biased genes in *G. mellonella*; we only found two genes (*GmelOBP19* and *GmelOR47*) that were mainly expressed in the male antennae. The male antennae-biased expression suggests that these genes may play a role in the recognition of volatiles from females and/or beehives. In other insect species, including *E. obliqua*, *O. furnacalis*, *Cotesia vestalis*, *Laodelphax striatellus*, *Leptocorisa acuta*, *Histia rhodope*, *Phthorimaea operculella*, and *C. medinalis*, a number of olfactory genes were also mainly expressed in male antennae (Zhang et al., 2015; Liu et al., 2017b, 2020; Sun et al., 2017; Li et al., 2020; Qu et al., 2020; Yang et al., 2020; He et al., 2021).

## Conclusion

In conclusion, this study generated a transcriptome dataset of *G. mellonella* antennae. From the dataset, we identified numerous olfactory genes, including 21 *OBP*s, 18 *CSP*s, 43 *OR*s, 18 *IR*s, and 2 *SNMP*s. Several genes displayed tissue- and sex-biased expression patterns, suggesting they may play a role in olfactory processes. These results, together with the data of Zhao et al. (2019) provide a resource for olfactory genes in *G. mellonella*. Future functional studies on these genes will provide greater understanding of the molecular mechanisms underlying *G. mellonella* olfaction.

## Data Availability Statement

The datasets presented in this study can be found in online repositories. The names of the repository/repositories and accession number(s) can be found below: https://www.ncbi.nlm.nih.gov/, SRR8307567 and SRR8307568.

## Author Contributions

X-CJ, SL, X-YJ, Z-WW, L-SY, and H-QC conceived and designed the experimental plan. X-CJ, SL, X-YJ, Z-WW, J-JX, QG, C-WS, T-FS, and H-RZ performed the experiments. X-CJ, SL, L-SY, and H-QC analyzed the data. X-CJ and SL drafted the manuscript. L-SY and H-QC refined and approved the final manuscript. All authors contributed to the article and approved the submitted version.

## Conflict of Interest

The authors declare that the research was conducted in the absence of any commercial or financial relationships that could be construed as a potential conflict of interest.

## Footnotes

1 https://blast.ncbi.nlm.nih.gov/Blast.cgi

2 http://www.ncbi.nlm.nih.gov/gorf/gorf.html

3 http://web.expasy.org/compute_pi/

4 http://www.cbs.dtu.dk/services/SignalP

5 http://www.cbs.dtu.dk/services/TMHMM/

6 http://www.ebi.ac.uk/tools/msa/clustalo/

7 http://tree.bio.ed.ac.uk/software/figtree/

8 http://meme-suite.org/tools/meme

9 https://www.ncbi.nlm.nih.gov/sutils/splign/splign.cgi

## Supplementary Material

Supplementary Figure 1 KOG classification of the *Galleria mellonella* unigenes.

Supplementary Figure 2 KEGG classification of the *G. mellonella* unigenes.

Supplementary Figure 3 A comprehensive list of *G. mellonella OBP*, *CSP*, *OR*, and *IR* genes.

Supplementary Figure 4 Partial alignment of the protein sequences of *G. mellonella* OBPs.

Supplementary Figure 5 Alignment of the deduced protein sequences of *G. mellonella* CSPs.

Supplementary Figure 6 Alignment of deduced *G. mellonella* SNMP protein sequences with orthologs from other insect species.

Supplementary Table 1 Accession numbers of OBPs, CSPs, ORs, IRs, and SNMPs used in phylogenetic analyses.

Supplementary Table 2 Amino acid sequences of OBPs and CSPs used in motif pattern analyses.

Supplementary Table 3 Primers used in this study.

Supplementary Table 4 Details of the olfactory genes identified in the *G. mellonella* antennal transcriptome.

Supplementary Table 5 Nucleotide and amino acid sequences of chemosensory genes identified in *G. mellonella*.

Supplementary Table 6 Details of the genomic distribution of the *G. mellonella* olfactory genes.
