# Supplementary material for: Identification of Olfactory Genes From the Greater Wax Moth by Antennal Transcriptome Analysis
Source: Front Physiol. 2021 May 19;12:663040. doi: 10.3389/fphys.2021.663040 (PMC8172125; doi:10.3389/fphys.2021.663040)

**Figure S1.** KOG classification of the *Galleria mellonella* unigenes.


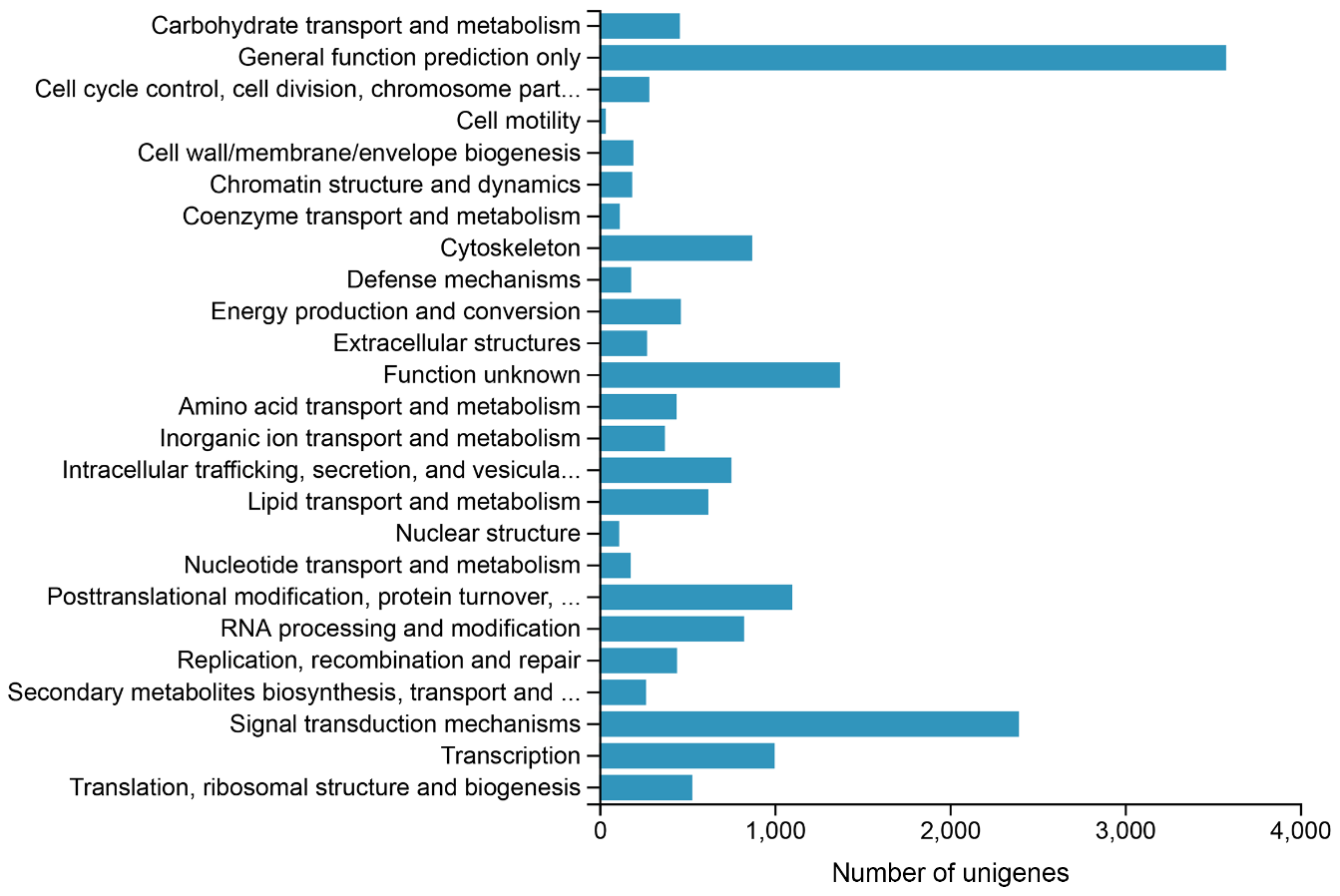


**Figure S2.** KEGG classification of the *G. mellonella* unigenes.


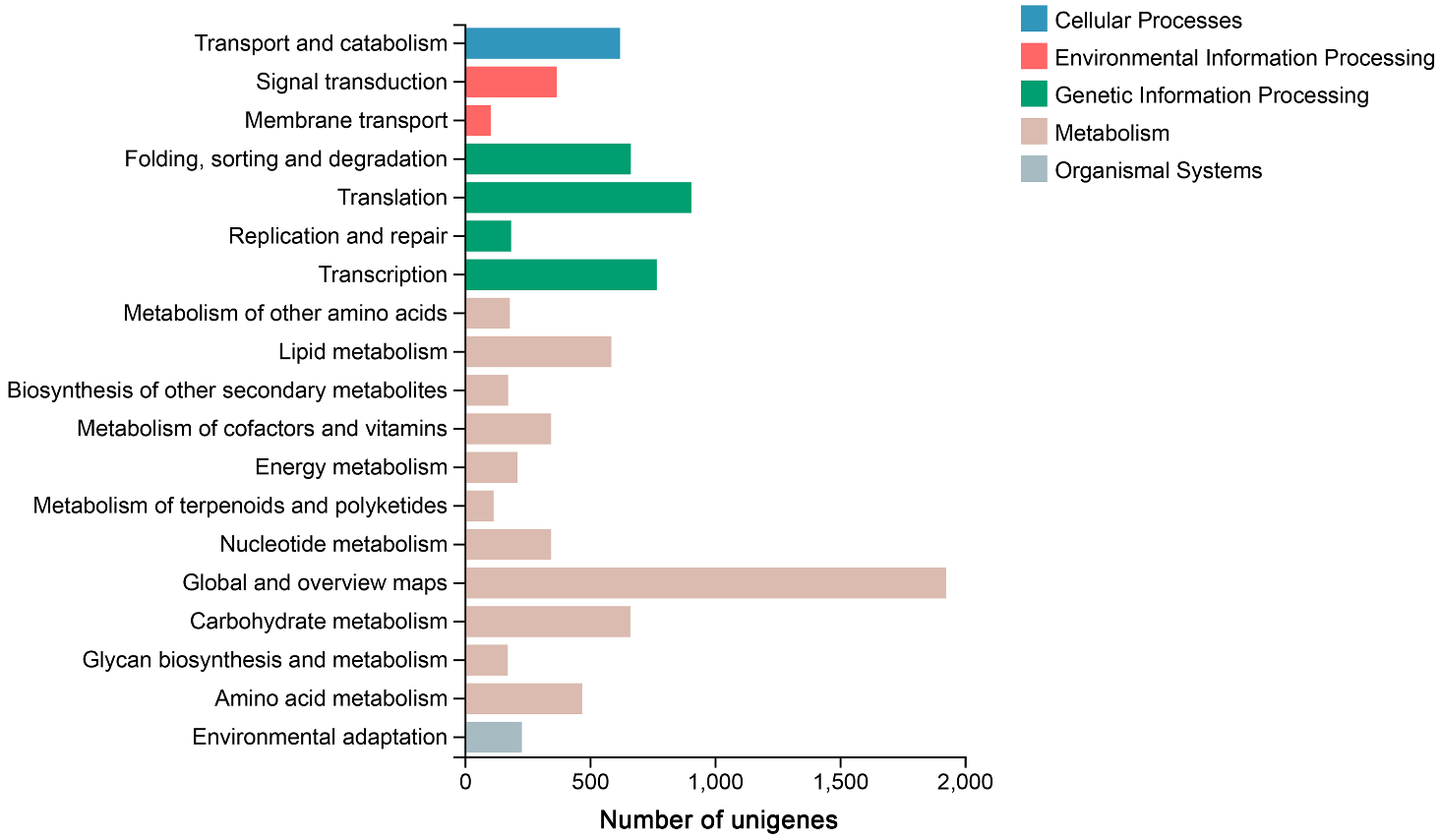


**Figure S3.** A comprehensive list of *G. mellonella* *OBP*, *CSP*, *OR* and *IR* genes. Genes identified by Zhao *et al*. (2019) and in this study are shown in red and blue boxes, respectively.

**Figure S4.** Partial alignment of the protein sequences of *G. mellonella* OBPs. Six positionally conserved cysteines are shaded in yellow and marked with Arabic numbers 1 to 6.


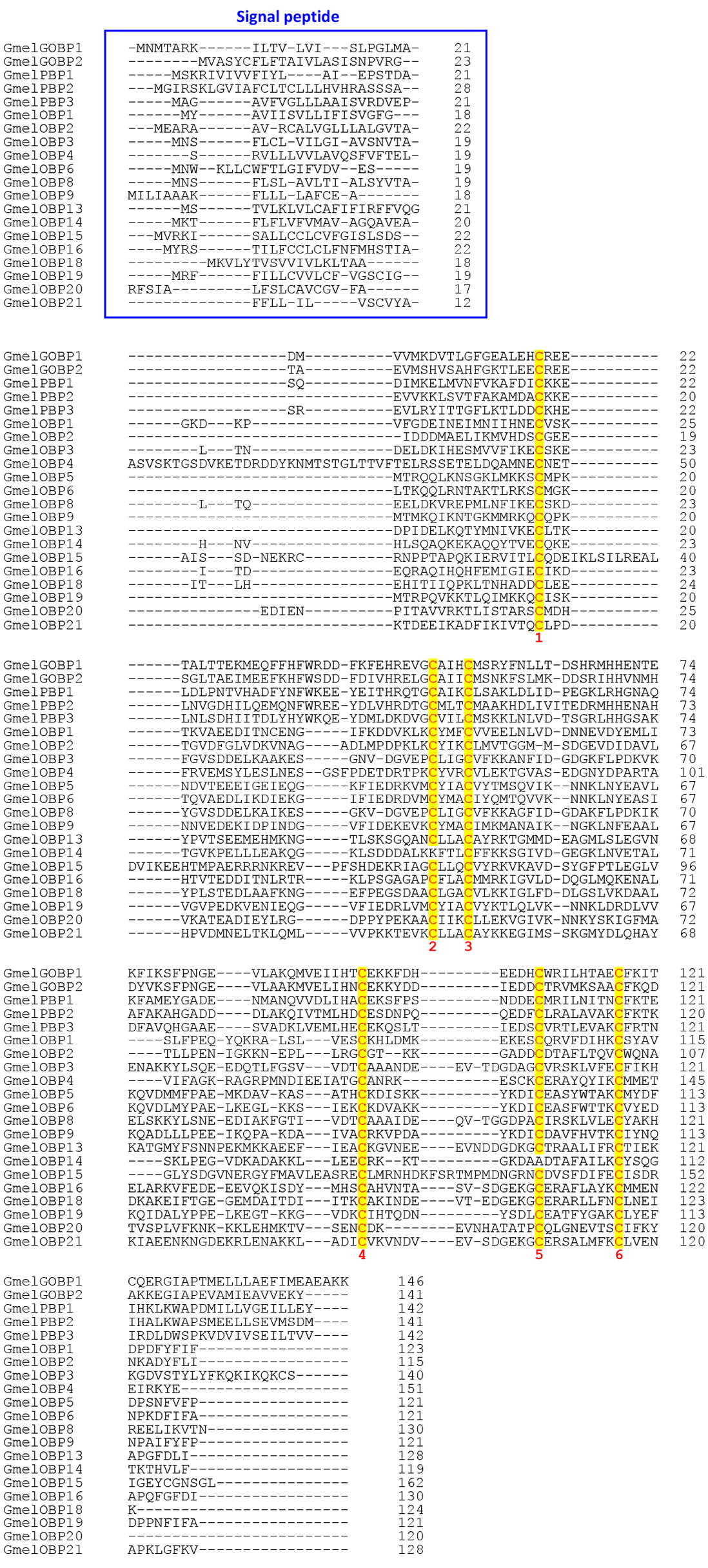


**Figure S5.** Alignment of the deduced protein sequences of *G. mellonella* CSPs. Positionally conserved cysteine residues are highlighted with yellow color and marked with Arabic numbers 1–4.


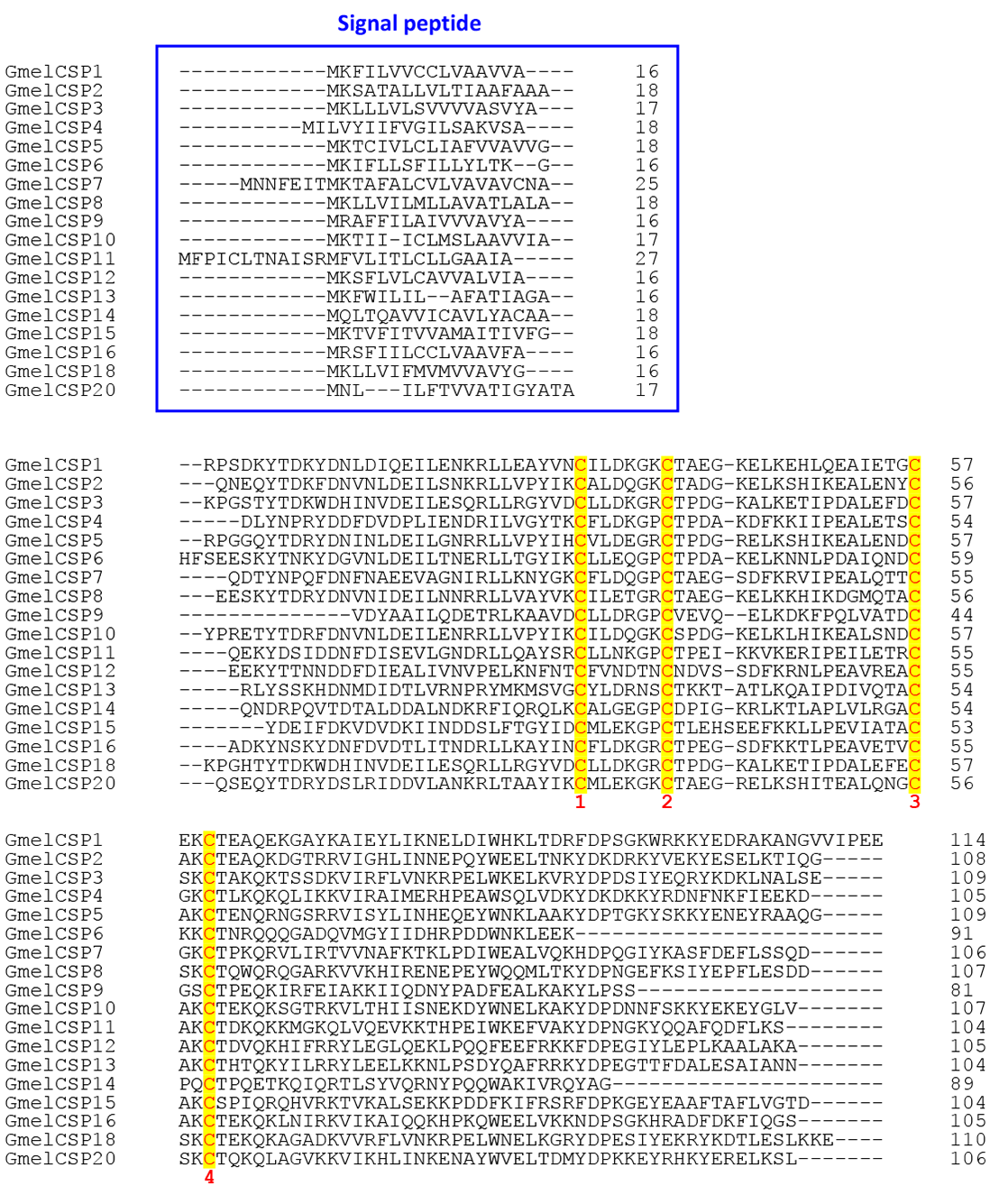


**Figure S6.** Alignment of deduced *G. mellonella* SNMP protein sequences with orthologs from other insect species. Abbreviations: Gmel: *G. mellonella*, Bmor: *Bombyx mori*, Hvir: *Heliothis virescens*, Cmed: *Cnaphalocrocis medinalis*, Csup: *Chilo suppressalis*, Ofur: *Ostrinia furnacalis*. The predicted transmembrane domains are underlined. Positionally conserved cysteine residues are highlighted with yellow color and marked with Arabic numbers 1–5.


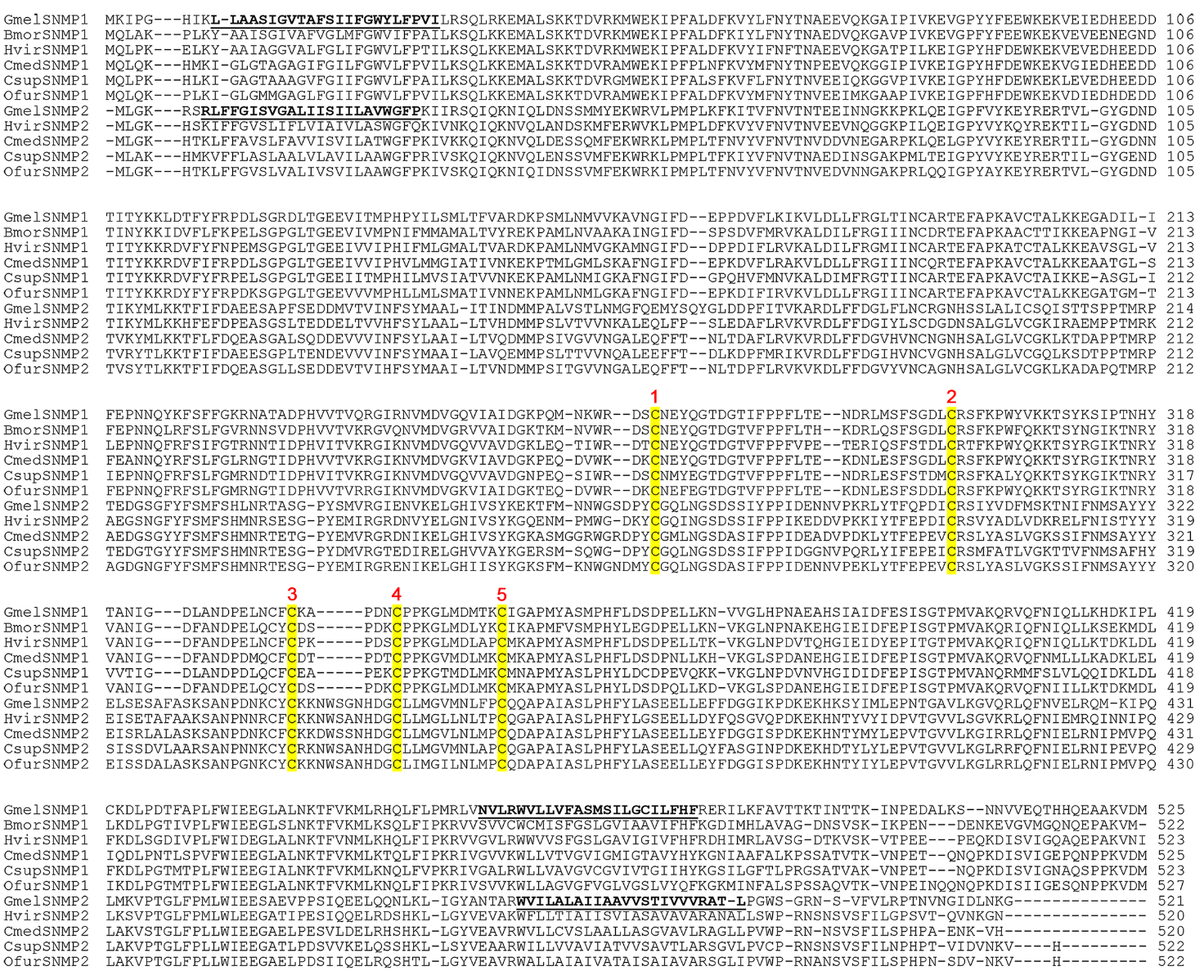

Supplement: Supplementary Figure 1 — KOG classification of the Galleria mellonella unigenes. [file Data_Sheet_1.DOCX]
